# Power of Digital Economy to Drive Urban-Rural Integration: Intrinsic Mechanism and Spatial Effect, from Perspective of Multidimensional Integration

**DOI:** 10.3390/ijerph192315459

**Published:** 2022-11-22

**Authors:** Zhengxin Li, Chengjun Liu, Xihui Chen

**Affiliations:** 1 Institute of Industrial Economics, Zhejiang University of Technology, Hangzhou 310023, China; 2 School of Management, Zhejiang Shuren University, Hangzhou 310015, China; 3 School of Management, Zhejiang University of Technology, Hangzhou 310023, China

**Keywords:** digital economy, urban-rural integration, multidimensional integration, spatial effect, geographically and temporally weighted regression

## Abstract

The consensus that the digital economy drives urban-rural integration has been gradually reached both in practice and theory. Besides, the way by which the digital economy drives urban-rural integration remains updated iteratively. The coming period is an important opportunity to break down the dualistic urban-rural structure and improve the urban-rural integration development. It is also a critical stage for China to promote the deep integration of the digital economy and the real economy. In this study, the intrinsic mechanism of the digital economy in driving the four dimensions of urban-rural integration was elaborated. An analysis was made of the spatial effects in 30 provinces (municipalities and autonomous regions) of China during 2011–2019 using Bivariate Global Moran’s I and geographically and temporally weighted regression (GTWR) models. As revealed by the results: (1) the digital economy and the four dimensions of urban-rural integration advance steadily, in which the convergence degree of urban and rural resident consumption is comparatively higher; (2) there is a significant spatial auto-correlation between the digital economy and the four dimensions of urban-rural integration, with the influence gradually strengthened with time; (3) the digital economy exerts mainly positive impacts on the equivalent allocation of urban and rural factors, integration of three industries in urban and rural areas, and convergence degree of urban and rural resident consumption, but inhibits the equalization of urban and rural public services in nearly half research areas; (4) both digital equipment basis and user basis play a vital role in promoting the four dimensions of urban-rural integration.

## 1. Introduction

National modernization is a process of continuously transforming and rebuilding the urban-rural relationship. As social development evolves, the urban-rural relationship in China has experienced a process of binary segmentation, binary icebreaking, and integration. Against the backdrop of a new economic pattern and journey, the following obvious changes have taken place in urban-rural structural features: China’s per capita gross domestic product (GDP) grew from 1400 USD in 2001 to 11,400 USD in 2020. When the income of urban and rural residents increased, their consumption structure also changed dramatically, and the Engel coefficient difference between urban and rural residents decreased from 9.5% in 2001 to 3.5% in 2020. As the urbanization level exceeds 60%, the total growth rate of migrant workers gradually slows down, and a new phenomenon of two-way flow acceleration of urban and rural resources is happening [1]. However, in the process of rapid urbanization, there exist problems such as the imbalance in the industrial structure between urban and rural areas [2], the restriction of the free flow of elements, and significant regional differences [3]. Meanwhile, with further development of a new round of scientific and technical revolution and industrial transformation, the digital economy is transforming the logic in the division of labor, optimizing resource allocation, and promoting the integration of urban and rural factors by virtue of its features of high substitutability, permeability, and inclusiveness, it plays an important role in building a new type of urban-rural relationship and promoting the rapid development of urban-rural integration. The coming period is an important opportunity to break down the dualistic urban-rural structure and improve the institutional mechanism for the integrated development of urban and rural areas. It is also a critical stage for China to promote the deep integration of the digital economy and the real economy and to continuously strengthen, improve and expand China’s digital economy. However, the digital economy in China’s urban and rural areas is seriously divided, and the digital divide still exists, which has become a prominent shortcoming in the integrated development of urban and rural areas in the new development stage. Therefore, China urgently needs to explore the intrinsic mechanisms and specific effects of the digital economy to drive urban-rural integration. In this context, this research is forward-looking.

As the digital economy develops rapidly and the connotation of urban-rural integration is constantly enriched, the approach by which the digital economy drives urban-rural integration remains updated iteratively. This requires a thorough and well-rounded understanding of the intrinsic mechanisms of the digital economy in driving urban-rural integration and further consideration of the spatial relationship between the digital economy and urban-rural integration. This research proposes hypotheses in three aspects. Firstly, the digital economy can promote urban-rural integration in four dimensions, respectively the equivalent allocation of urban and rural factors, the integration of three industries in urban and rural areas, the equalization of urban and rural public services, and the convergence of urban and rural resident consumption. Secondly, there are spatial differences in the impact of the digital economy on urban-rural integration, especially the differences between east China, central China, and west China. Thirdly, the influence of each subdivided kinetic energy of the digital economy on urban-rural integration is different.

Therefore, this research first proposes that the digital economy drives urban-rural integration in four dimensions, namely, the equivalent allocation of urban and rural factors, the integration of three industries in urban and rural areas, the equalization of urban and rural public services, and the convergence of urban and rural resident consumption, and uses this as a framework to construct and improve the indicator evaluation system of the digital economy and urban-rural integration. Secondly, a bivariate spatial autocorrelation model is used to preliminarily explore whether there is a spatial correlation between the digital economy and urban-rural integration. On this basis, the geographically and temporally weighted regression (GTWR) model is used to further explore the spatial effects of the digital economy on urban-rural integration in both time and space dimensions. Finally, this research is expected to provide theoretical support and policy suggestions for the digital economy and urban-rural integrated development to deeply explore the value and functions of the digital economy and promote high-quality urban-rural integrated development and urban-rural common prosperity.

## 2. Literature Review

The literature review is represented in three parts: the definition of the digital economy, the definition of urban-rural integration, and the studies of the combination of the digital economy and urban-rural integration. The concept of the digital economy was formally introduced by Don Tapscott in 1994 with the publication of the book “The Digital Economy” [4]. Most scholars consider the digital economy an economic activity [4,5,6,7,8,9]. It can change people’s work and life, driving economic development while reducing environmental impact, and is a new type of economic form [4,5]. Considering the broad connotation of the digital economy, it is the economic form in which goods and services are traded through digital technology [6]. The G20 Summit released by China in 2016 proposed a definition of the digital economy, which states that the “digital economy refers to the use of digital information and knowledge as factors of production, information technology network as a carrier, and ICT to promote efficiency improvement and macroeconomic structure optimization the sum of economic activities.” Besides, there are two main types of approaches to measuring the digital economy by institutions and academia. The first approach is the absolute value method, which counts and estimates the total size of digital economy-related industries in a region. The second approach is the relative value method, which constructs a multidimensional indicator system and standardizes it to compare the digital economy of different regions. For example, the Organization for Economic Cooperation and Development (OECD) constructs the digital economy in four dimensions: intelligent infrastructure, innovation capacity, digital industry (ICT for economic growth and job creation), and empowered society [7], and the European Union (EU) published the Digital Economy and Society Index (DESI), which measures the digital economy by four first-level indicators: human capital, internet connectivity, commercial application of digital technology, digital public services [8]. Hanna (2020) built an evaluation index for the digital economy in terms of digital infrastructure, digital platforms, digital governance, and data processing [9].

The concept of urban-rural integration was first put forward by Engels in the 1840s, who considered it the last stage in the process of the development of urban-rural relations [10]. Many researchers hold the opinion that urban-rural integration is manifested in the resource free flows of capital, material, technology, labor force, and land between urban and rural areas in general [11,12,13]. Besides, urban-rural integration can also represent the urban and rural industries developed together [14,15]. Due to the wide scope of the concept, there is no unified measurement of urban-rural integration. Chen et al. (2020) built the theoretical framework of urban-rural integration in three aspects, the convergence of elements flows such as population and capital, urban-rural equivalence consists of equal work with equal pay, coordinated development including new-type industrialization, and agricultural modernization [16]. Yang et al. (2021) built a multidimensional evaluation index system of urban-rural integration. Firstly, the free flow of urban and rural land, capital, population, and technology are the basis, and the interaction of industries is the premise. Secondly, the transportation information network is the internal driving force of urban-rural integration. Thirdly, the goal is the equivalence of public services and the equalization of quality of life for residents in urban and rural areas [17]. It is also pointed out that urban-rural integration corresponds to the coordination of economic and social development in urban and rural areas, the innovation of a combination of urban and rural elements, the improvement of the marketization degree of urban and rural factors, the optimization of urban and rural ecological environment and spatial layout, and the equal sharing of urban and rural development achievements [18,19].

Currently, the consensus that the digital economy drives urban-rural integrated development has been gradually reached both in practice and theory. “Digital” as a concept and a factor has been introduced in the research of urban-rural integration successively by academia. The studies of the combination of the digital economy and urban-rural integration can be divided into two types: theoretical discussion and empirical analysis. For the theoretical discussion, it is indicated that the digital economy can promote migrant economic integration in urban and rural areas but inhibit their psychological integration and sociocultural integration [1]. It is concluded that digital infrastructure quality in urban and rural areas has a growing and persistent difference, and the policies to narrow the digital infrastructure gap between urban and rural areas are essential [20,21]. Besides, some scholars hold the opinion that producers can remold and integrate three industries in urban and rural areas and create new industry statuses and patterns by adopting the internet, artificial intelligence, and other technologies in the digital economy [22,23]. Moreover, network platforms can accelerate the matching and circulation of various commodities and data as new production factors are further changing the income distribution structure to narrow the urban-rural income gap [24]. It was also found that new consumer goods can be produced through digital technologies, promoting consumption upgrades and transformation and expanding consumption demands [25]. In addition, digital governance can address the problem of “information isolated islands,” reduce transaction costs, realize the urban-rural interconnection, and promote the high-quality development of public services [26]. Another researcher summarized four effects of the digital economy on the urban-rural structure transformation, namely factor allocation effects, product supply effects, income distribution effects, and social governance effects. Besides, the digital economy enables the two-way flow of urban and rural factors, industrial transformation and upgrade, public goods allocation adjustment, and geographical space reconstruction [27]. In the empirical analysis, many researchers investigated the influences of digital inclusive finance (a sub-topic of the digital economy) on urban-rural resident income and consumption gaps (sub-topics of urban-rural integration) [28,29,30]. They verified the positive role of digital inclusive finance in narrowing the urban-rural income or consumption gap.

Most of the extant research focuses on the definition and measurement of the digital economy or urban-rural integration. However, studies of the combination of the two concepts are lacking. Although some scholars have explained the mechanism of the digital economy driving urban-rural integration, few scholars have empirically tested the relationship between the two concepts. The empirical studies only lie in the influences of digital inclusive finance on urban-rural resident income and consumption gaps. Therefore, this paper aims to fill the research gap from two aspects: (1) complementing and improving the intrinsic mechanism of the digital economy to drive urban-rural integration. (2) Conducting an empirical analysis of the digital economy to drive urban-rural integration from a multidimensional perspective, especially the analysis of spatial effect.

## 3. Intrinsic Mechanisms

**Figure 1:**
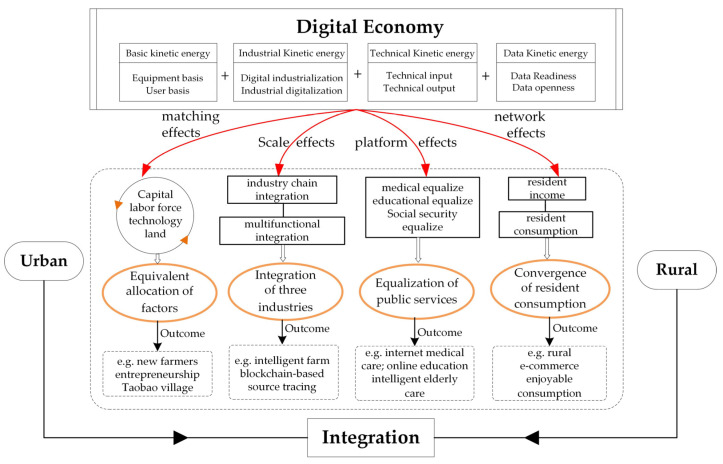
Intrinsic mechanisms.

The intrinsic mechanism refers to the way the digital economy drives urban-rural integration. It is a detailed theoretical framework built according to previous research. Digital economy-driven urban-rural integration represents a complicated relationship between two multilayer and multifactor systems. As for two such systems, a digital economy featuring high permeability and non-competitiveness can drive the urban-rural integration in depth through platform effects, scale effects, long tail effects, substitution effects, and network effects achieved by changing the logic in the traditional division of labor, reforming the resource allocation model, breaking the boundaries of time and space, lowering transaction costs, and reducing the information asymmetry. With reference to the main opinions in the existing literature [1,20,21,22,23,24,25,26,27,28,29,30], it was proposed in this research that the digital economy stimulated urban-rural integration in four dimensions, namely equivalent allocation of urban and rural factors, integration of three industries in urban and rural areas, equalization of urban and rural public services, and convergence of urban and rural resident consumption (Figure 1).

### 3.1. Equivalent Allocation of Urban and Rural Factors

The digital economy drives the equivalent allocation of urban and rural factors, including capital, labor force, technologies, land, and other factors. For capital, capital allocation to rural areas is encouraged by digital inclusive finance, and financial risks can be controlled by digital means. As to the labor force, effectively reduced information asymmetry, enhanced matching degree of supply and demand of the labor force, and accelerated flow of urban and rural labor force are achieved by digital economy via matching effects. Further, the labor productivity of some rural industries can also be improved, resulting in the backflow of the migrant labor force and even of the urban labor force (returning home for entrepreneurship) [18]. As a result, the dynamic equilibrium of urban and rural labor force can be gradually realized. For technologies, labor force mobility also involves talent flow and thus causes the transformation of scientific research achievements after returning home. In terms of land, the scale and range of land transfer are increased due to the decrease in transaction costs caused by progress in digital technologies. Supported by the digital economy, the above-mentioned factors will be gradually transformed from “one-way flow” to “two-way flow” between urban and rural areas [19].

### 3.2. Integration of Three Industries in Urban and Rural Areas

The digital economy stimulates the integration of three industries in urban and rural areas, including two types of integration. From the angle of industry chain integration, the digital economy permeates the production, processing, sales, and other links of urban and rural industries, forming the basic industry models of digital production, digital logistics, and digital marketing [31]. For instance, the production link of the supply side covers intelligent farms and intelligent agricultural machinery, the logistics link covers the blockchain-based source tracing and logistics cloud muster house, and the digital marketing of the demand side covers digital exhibition and brand marketing. From the perspective of multifunctional integration, the digital economy exerts scale effects and range effects, thus allowing for its deep cross-integration with agricultural, cultural, and tourism industries [32]. For example, a global intelligent tourism platform can be established on the basis of the digital economy.

### 3.3. Equalization of Urban and Rural Public Services

The digital economy motivates the equalization of urban and rural public services, which involve education, medicine, elderly care, etc. On the one hand, the digital economy can reduce information asymmetry, lower transaction costs, and blur the range boundary. In addition, it can promote platform development for and diversification of the subjects, models, and application scenes of public service products [33]. In this process, the equalization of urban and rural public services is motivated by the digital economy through such resultful measures as internet medicine, online education, and intelligent elderly care. On the other hand, demand information can be precisely summarized by the digital economy via platform effects and long-tail effects. The overall coordination function is conducive to promoting the precise supply of urban and rural public service products, standardization of urban and rural public services, and remote sharing of urban and rural public services [34].

### 3.4. Convergence of Urban and Rural Resident Consumption

The digital economy drives the convergence of urban and rural resident consumption. On the one hand, the digital economy has strong network effects, and digital platforms allow for equal options for commodity consumption for urban and rural residents [29,30]. On the other hand, the consumption structure upgrade is indirectly mobilized by the digital economy via the enhancement of the urban and rural resident income, and the digital economy stimulates the income increase of rural residents in an effective manner through rural e-commerce and intelligent agriculture. Hence, the transformation from rural survival consumption to developmental and enjoyable consumption is achieved, gradually converging urban and rural resident consumption habits.

## 4. Index Development, Data Sources, and Methods

### 4.1. Development of Index

Based on the aforesaid interpretation of the intrinsic mechanisms and relevant research results [18,19,20,21,22,23,24,25,26,27,28,29,30,31,32,33,34], urban-rural integration was measured in four dimensions, namely equivalent allocation of urban and rural factors, integration of three industries in urban and rural areas, equalization of urban and rural public services, and convergence of urban and rural resident consumption in this research. Given the availability of data regarding the equivalent allocation of urban and rural factors, the allocation of four factors, including the capital, labor force, technologies, and land, was measured. Two types of integration were measured for the integration of three industries in urban and rural areas. In terms of equalization of urban and rural public services, medical, educational, and social security equalization was measured. For the convergence of urban and rural resident consumption, resident income and consumption were measured. Moreover, “rural data/urban data” was employed as an urban-rural ratio index (except for the “urban-rural Engel coefficient”). The degree of closeness of indices to 1 was in proportion to the closeness between rural and urban data. In this way, a multidimensional index system of urban-rural integration was developed (Table 1, where all indices were positive).

In terms of measurement of the digital economy, based on the existing research approach [4,5,6,7,8,9] as well as the definition of digital economy at the G20 Summit and relevant statement in Statistical Classification of Digital Economy and Its Core Industries released by the National Bureau of Statistics in China, the digital economy was evaluated from four dimensions, including basic kinetic energy, industrial kinetic energy, technical kinetic energy, and data kinetic energy. Basic kinetic energy is a foundation of the digital economy and a crucial guarantee for narrowing the urban-rural “digital divide,” which was divided into equipment basis and user basis in this research. Industrial kinetic energy is a core power of the digital economy, including digital industrialization and industrial digitalization. The former was characterized by the total number of telecom services and the total output value of digital industries (manufacturing industry and service industry). This research characterized the latter by e-commerce sales and digital inclusive finance. Technical kinetic energy is a key support of the digital economy, measured from perspectives of technical input and output in this research. Data kinetic energy is an important bridge of the digital economy in which data serve as the critical production factor. Data valuation relies on the market-oriented allocation of data. As a result, data kinetic energy was measured in data readiness and openness, and an overall index system of the digital economy was built in this research (Table 2, where all indices were positive).

Given the different dimensions of each index, the extremum method was adopted in this research to standardize indices, and the weight was determined by subjective and objective methods (expert evaluation for the weight of second-grade indices and the entropy method for that of third-grade indices), so as to ensure the accuracy and rationality of calculation results.

### 4.2. Data Sources

Based on the integrity, availability, and scientific nature of data, a total of 30 provinces (municipalities and autonomous regions) except Tibet, Hong Kong, Macao, and Taiwan, China, were taken as research objects in this work, with provinces with a large amount of missing data eliminated. Considering that there were lots of missing data about the digital economy before 2011 and the rapid development in recent years, 2011–2019 was selected as the research period in this work. The data relating to the digital economy and urban-rural integration were derived mainly from the China Statistical Yearbook, China Rural Statistical Yearbook, China Social Statistical Yearbook, China Population & Employment Statistical Yearbook, and statistical yearbooks of provinces (municipalities and autonomous regions) from 2012 to 2020, and partial missing data were supplemented using interpolation.

### 4.3. Methods

#### 4.3.1. Bivariate Spatial Autocorrelation (Bivariate Global Moran’s I)

This research aims to explore the spatial effects of the digital economy driving urban-rural integration. Before discussing the specific effects, it is necessary to initially empirically test whether there is a spatial correlation between the digital economy and urban-rural integration within the entire geographical area. The first law of geography states that there is a correlation between everything and that the closer the distance, the stronger the correlation [35]. The traditional method to measure spatial correlation is Global Moran’s I [36,37,38]. It indicates overall clustering within the entire geographical area or the level of spatial autocorrelation by calculating a pseudo *p*-value. These are the result of complex permutation methods to determine significant differences between spatial units [39,40]. Univariate global Moran’s I use only one variable, for example, the digital economy and the spatially weighted value of the digital economy. Bivariate Global Moran’s I determine the direction and strength of the relationship between two variables, such as the digital economy and urban-rural integration in each dimension [41,42,43,44,45]. This approach provides preliminary information on the likely direction of the effect.

The specific calculation process [39,43,44,45] is as follows:

(1) $$I=\frac{\sum_{i=1}^{n}\sum_{j=1}^{n}W_{ij}(x_{i}-\bar{x})(y_{j}-\bar{y})}{\sigma^{2}\sum_{i=1}^{n}\sum_{j=1}^{n}W_{ij}}$$

where, I denotes the bivariate spatial autocorrelation index, n is the sample size, σ^2^ denotes the variance of all samples, and W_ij_ is the spatial weight matrix. Besides, x_i_ refers to an independent variable (the developmental level of the digital economy), y_j_ is the observed value of dependent variables (urban-rural integration level in various dimensions) in the spatial units i and j, and $\bar{x}$ and $\bar{y}$ refer to the average values of spatial observation.

The Bivariate Global Moran I model has been used by researchers worldwide. For example, Parenteau et al. (2011) analyzed the historical data of Canada to provide information on the strength of the associations between NO2 concentrations and the respiratory health outcome rate [46]. Lu et al. (2022) examine the spatial correlation between urban form centrality and land surface temperature (LST) by investigating 27 cases of Chinese megacities [42]. Rusche et al. (2011) adopted the model for the purpose of a bidimensional spatial analysis of the regional association between two German wood-based industries [47]. Loughnan et al. (2008) used the bivariate global Moran’s I to determine the strength and direction of the relationship between the standardized incidence ratio and the population’s age distribution in each local statistical area [48].

#### 4.3.2. Geographically and Temporally Weighted Regression (GTWR)

The previous method, Bivariate Global Moran’s I, determines whether there is a spatial relationship between the digital economy and urban-rural integration. If there is a spatial correlation between them, the next step is to explore the extent to which the digital economy affects urban-rural integration. Traditional spatial econometric models, for example, the spatial lag model (SLM), spatial error model (SEM), and spatial Durbin model (SDM), all take a global view to define variable relationships, and the regression coefficient is a fixed value for the whole research area, lacking local spatial difference. Therefore, in order to explore the differences between regions in greater depth, the geographically and temporally weighted regression (GTWR) model was put forward [49]. It can estimate the independent variable coefficients of different areas and represent the local spatial effects. As an extension of the GWR model [50,51], the GTWR model was figured out by adding the time dimension into the GWR model. The advantage of using the GTWR model is that spatial and temporal coordinates are added to calculate the space-time weight matrix to solve the nonstationarity of variables in the time and space dimensions effectively. The traditional GWR analysis does not introduce the temporal dimension [52].

In this research, the GTWR model was adopted to analyze the way by which the digital economy drives various dimensions of urban-rural integration in this research, specifically expressed below:

(2) $$Y_{i}=\alpha_{0}(u_{i},v_{i},t_{i})+\sum_{k}\beta_{k}(u_{i},v_{i},t_{i})X_{ik}+\varepsilon_{i}$$

where, (u_i_, v_i_, t_i_) is the time-space coordinate of samples in the ith area, and u_i_, v_i_, t_i_ is longitude, dimension, and time, respectively. Y_i_ presents the dependent variables, namely urban-rural integration in various dimensions, including equivalent allocation of urban and rural factors, integration of three industries in urban and rural areas, equalization of urban and rural public services, and convergence of urban and rural resident consumption, and the subscript i presents different sample observation points. X_ik_ stands for the observed value of the kth variable (including the independent variables and control variables) in the area sample i, and ε_i_ stands for the random error term. In this research, the GTWR model was employed to analyze the overall effects of the digital economy as a whole on various dimensions of urban-rural integration and visualization, followed by the effect decomposition of the digital economy as the independent variable. β_k_(u_i_, v_i_, t_i_) refers to the kth regression parameter of the ith sample. In accordance with the local weighted least squares method, the expression of estimating parameters is as follows:

(3) $$\hat{\beta }(u_{i},v_{i},t_{i})={\left[X^{T}W(u_{i},v_{i},t_{i})X\right]}^{-1}X^{T}W(u_{i},v_{i},t_{i})Y$$

where, β_k_(u_i_, v_i_, t_i_) is the estimated value of β_k_(u_i_, v_i_, t_i_). X is the matrix of independent variables, X^T^ expresses the transposition of the matrix, Y stands for the matrix of dependent variables, and W (u_i_, v_i_, t_i_). expresses the time-space coordinate weight matrix. The gaussian distance function was adopted in this work. The time-space weight matrix was obtained via the bi-square space weight function. The time-space distance between the area samples i and j was expressed as follows:

(4) $$d_{ij}=\sqrt{\delta [{\left(u_{i}-u_{j}\right)}^{2}+{\left(v_{i}-v_{j}\right)}^{2}]+\gamma {\left(t_{i}-t_{j}\right)}^{2}}$$

Herein the optimum bandwidth needed to be determined to improve the accuracy of the model, and the self-adaptive bandwidth was chosen in this research to determine the time-space weight.

The GTWR model is also widely used by scholars. Ling et al. (2022) used the data in the US to explore the influencing factors of human activities and socio-demographics during the COVID-19 pandemic by the GTWR model [53]. Wang et al. (2022) used the GTWR model to research urban expansion patterns and their driving forces by taking the Beijing-Tianjin-Hebei (Jing-Jin-Ji) urban agglomeration as an example [54]. Mirzaei et al. (2022) utilized the GTWR model to investigate the spatial and temporal variability relationship between satellite aerosol optical depth (AOD) data and PM2.5 concentrations measured at ground monitoring stations in Tehran, Iran [55].

## 5. Results and Discussions

### 5.1. Process Analysis on Digital Economy and Multidimensions of Urban-Rural Integration

**Figure 2:**
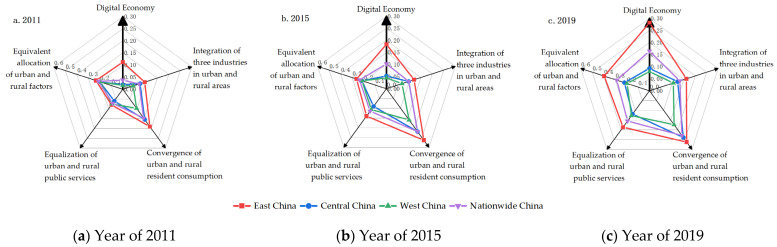
Radar graph.

After the digital economy and four dimensions of urban-rural integration were subjectively and objectively empowered respectively and standardized, a radar graph was drawn via Origin software (Figure 2), of which the center denoted the origin and the vertex represented the maximization. The maximization of the digital economy was 0.30, and that of the four dimensions of urban-rural integration was 0.60. The digital economy and the four dimensions of urban-rural integration were analyzed, with 2011, 2015, and 2019 from 2011–2019 selected as representative years.

According to the results, (1) in terms of the numerical values, east China had the highest development level of the digital economy, followed by central and western China, and for the four dimensions of urban-rural integration, the convergence of urban and rural resident consumption was at the highest level. Besides, the equalization of urban and rural public services in west China exhibited a special performance, showing an “east-west-central China” declining pattern. Such a result, different from the “east-central-west China” declining trend of the other three dimensions of urban-rural integration, is consistent with the research conclusions of Ma et al. (2018) [56]. (2) In terms of the annual growth rate, the digital economy and the four dimensions of urban-rural integration were growing nationwide. The average annual growth rate of the development level of the digital economy was 36.98% in the past nine years, and the four dimensions of urban-rural integration had average annual growth rates of 3.83%, 9.61%, 14.73%, and 6.23%, respectively. This suggested that the digital economy has developed rapidly in the past nine years, with a growth rate higher than that of the four dimensions of urban-rural integration. By region, west China exhibited the highest average annual growth rate in both the digital economy and urban-rural integration, similar to the research results of Zhao et al. (2022) [57].

### 5.2. Bivariate Global Moran’s I

Based on the above-mentioned preliminary evaluation, the changing trend of the digital economy was relatively similar to that of the four dimensions of urban-rural integration in the three regions (east, central, and west China), and there existed certain spatial synchrony between the regions with a high development level of the digital economy and those with good urban-rural integration. In order to further verify the correlation between them, the evolution features of their spatial correlation were explored via bivariate Global Moran’s I. Geoda software was used to calculate such bivariate Global Moran’s I (Table 3). In general, the digital economy basically displayed a significant positive spatial correlation to the four dimensions of urban-rural integration, indicating an obvious spatial correlation between the digital economy and urban-rural integration.

The horizontal comparison (comparison of various dimensions) significantly changed the bivariate Global Moran’s I. The spatial correlation between the digital economy and convergence of urban and rural resident consumption was higher than those of the digital economy with an equivalent allocation of urban and rural factors, integration of three industries in urban and rural areas, and equalization of urban and rural public services. According to the vertical comparison (comparison of representative data in 2011, 2015, and 2019), the bivariate Global Moran’s I of each dimension gradually increased. That is, the positive spatial correlation of digital economy with the four dimensions of urban-rural integration was gradually strengthened, which is attributed to the implementation and continuous promotion of rural revitalization, new urbanization, digital rural development, and urban-rural integrated development strategies.

### 5.3. Spatial Effects of the Digital Economy in Driving Urban-Rural Integration

#### 5.3.1. Variable Description

Based on the bivariate Global Moran’s I, a significant positive spatial link existed between the digital economy and the four dimensions of urban-rural integration in the whole region. Therefore, in this study, the GTWR model was used to continue exploring the digital economy’s local spatial effects on the four dimensions of urban-rural integration, with the digital economy as the core explanatory variable and the four dimensions of urban-rural integration as the explained variables. In addition, relevant control variables were introduced into the model to alleviate the endogenous problems caused by other variables. According to relevant research [22,23,24,28], the following control variables were selected: gross domestic product (GDP) (gdp) for economic development measurement, the value of regional import and export (open) for opening up measurement, government expenditure (gov) for government supply measurement and regional relief amplitude (topogra) for natural conditions measurement (Table 4). Besides, all variables were standardized.

#### 5.3.2. Spatial Effect Analysis

To further identify the applicability of the GTWR model, the GTWR, GWR, and temporally weighted regression (TWR) models were separately employed for data fitting. The model parameters are listed in Table 5. As to the four explanatory variables, the residual sums of squares (RSS) and sigma values of the TWR model were significantly greater than those of the GTWR and GWR models. In contrast, the adjusted R2 of the TWR model was smaller than that of the GTWR and GWR models. Besides, in the TWR model, explanatory variables (2) and (4) failed the 10% significance test, suggesting relatively poor applicability of the TWR model. Then, all parameters of the GTWR model were slightly better than those of the GWR model, signifying that the influences of the digital economy on the four dimensions of urban-rural integration are heterogeneity in time and space, and the GTWR model possesses more analytical advantages.

**Figure 3:**
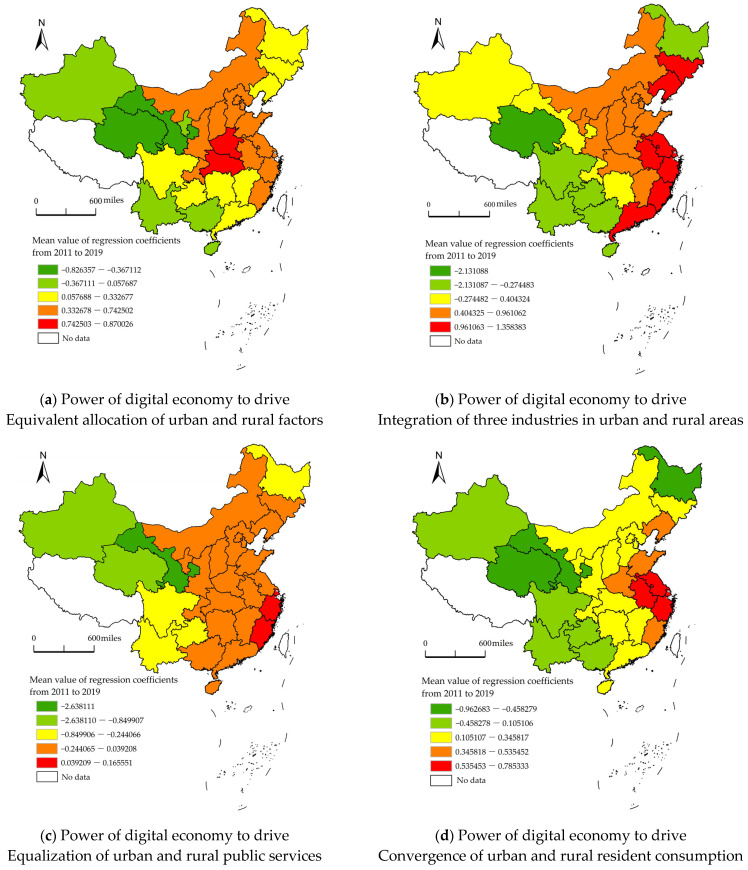
Estimated distribution of the regression coefficients of the GTWR model for the influences of the digital economy on the four dimensions of urban-rural integration. (Drawing review number: GS (2020) 4618 (supervised and manufactured by the Ministry of Natural Resources of the People’s Republic of China). It is a base map without modification, in which data from Tibet, Hong Kong, Macao, and Taiwan are excluded).

The regression coefficients of the GTWR model were visualized (Figure 3). It was revealed that the effects of the digital economy on the four dimensions of urban-rural integration exhibited flake-like and band-like distribution features, and the regression coefficients had both positive and negative effects in all dimensions.

(1) Equivalent allocation of urban and rural factors was promoted by the digital economy in most regions. During the research period, the strongest positive effect was observed in Henan and Hubei in central China, which gradually spread to the surrounding areas and weakened. The positive effect in east China was relatively strong. However, in northwest and southwest China, there was evident inhibition on the equivalent allocation of urban and rural factors by the digital economy. On the one hand, the digital economy improves employment modes and employment structures. It provides more employment opportunities by breaking the barrier of factor flow, thus driving the gradual backflow of the labor force, capital, and technologies from east China to central and west China. In central China, returning home for entrepreneurship is particularly prominent, and counties, as an important carrier of China’s urban-rural integration, undertake most of the returning labor force. The result is similar to the research conclusion of Zou et al. (2022) [1]. On the other hand, an inverted “U” shape relation may be found between the digital economy and urban and rural factor allocation. In other words, the digital economy in central China is at the initial stage of development, showing an obvious influence on urban and rural factor allocation, whereas the influences of the digital economy in east China on urban and rural factor allocation begin to fall back into the second half of the inverted U shape after reaching a certain degree since the digital economy in east China has developed earlier with too much factor agglomeration. Besides, the digital economy in Xinjiang, Qinghai, Ningxia, Gansu, Guangxi, and Yunnan in the northwest and southwest of China has a negative influence on urban and rural factor allocation, with the factors in these regions still in a state of net outflow, displaying a “siphon effect” of such factors in central and east China, thereby inhibiting the equivalent allocation of urban and rural factors.

(2) The effect of the digital economy on the integration of the three industries in urban and rural areas presented a band-like distribution increasing from west to east, with the high values of regression coefficients mainly concentrated in the east coastal region of China. The integration of the three industries in urban and rural areas has been achieved by the digital economy through industry chain integration and multifunctional integration. Some scholars believe that such complex and challenging integration is still in its infancy and expansion periods from the angle of the life cycle theory [2]. Thus, excellent institutional support and guarantees are required, including the optimization of industrial structures, guarantee of infrastructure, popularization of e-commerce logistics, support by financial systems, foundations for economic development, guidance by policies and systems, and improvement of legal systems. The institutional support and guarantee systems in the eastern coastal region of China are more complete than those in central and western China. Thus, the digital economy can play a better role in extending industry chains, including production, supply, and sale chains, in the eastern coastal region of China. In addition, the eastern coastal region of China also has a relatively higher multifunctional reticulate integration degree than central and west China. Dou et al. (2022) also indicate regional heterogeneity in the effect of the digital economy on industry chain integration [58].

(3) A difference was also observed in the effect of the digital economy on the equalization of urban and rural public services between east China and west China. A negative effect on the equalization of urban and rural public services was found in 13 regions, far more than the number of regions where the other three dimensions (equivalent allocation of urban and rural factors, integration of three industries in urban and rural areas, and convergence of urban and rural resident consumption) were negatively affected by the digital economy. Meanwhile, the equalization of urban and rural public services had the lowest absolute value of the negative number (−2.638) among the four dimensions, revealing the most obvious regional differences in the equalization of urban and rural public services in urban-rural integration. Theoretically, the digital economy can reduce the costs and improve the proceeds of public services, attracting high-level talents and further improving the development level of digital economy. As a result, a positive feedback mechanism is formed. However, such a mechanism further widens the regional differences so that the digital economy has an increasingly obvious inhibition of the equalization of urban and rural public services in central and west China. Moreover, the “digital divide” caused by uneven digital access, differences in skills used, and inequality in using effects is also an important reason for the inequality of urban and rural public services. Comparatively speaking, digital public services in negative-value regions have preferential effects in urban areas. In comparison, there are endowment differences between rural and urban areas in terms of educational and medical gaps. Therefore, the inequality of digital public services is further deepened.

(4) The digital economy stimulated the convergence of urban and rural resident consumption. The digital economy positively affected the convergence of urban and rural resident consumption gradually from east to west, with a particularly significant positive effect in the Yangtze River Delta. As for northwest and southwest China, there was a negative influence. It is partially because the influences of the digital economy on the consumption of urban and rural residents lie in the transaction costs, consumption modes, and consumption habits. In regions with high-level economic development and urbanization, a lot more digital economic resources have been allocated to rural areas. The rural residents in economically developed regions are also well educated, so it is easier for them to change their consumption habits and accept and apply new digital consumption modes. As a result, the digital economy can promote the convergence of urban and rural resident consumption. Contrarily, in economically backward regions, the digital economy brings more obvious consumption upgrades in cities and towns. A growing gap exists in consumption modes and habits between rural and urban residents because of the “digital divide.” It shares the same view with Luo et al. (2022) [30] and He et al. (2022) [59]. As revealed in Figure 3, the digital economy inhibited the convergence of urban and rural resident consumption in northwest and southwest China and widened the gap between urban and rural consumption in some regions. In addition, the digital economy displayed the greatest positive influence on urban and rural resident consumption in the Yangtze River Delta, which is closely related to the development strategy of the regional integration of the Yangtze River Delta in recent years. The regional integration process is deeply engaged in urban and rural areas in various regions so that urban and rural areas are interconnected with and dependent on each other and form an organic whole. Additionally, the regional integration process does have an important role in promoting the convergence of urban and rural resident consumption by the digital economy.

#### 5.3.3. Spatial Effect Decomposition

To further explore the influence of each subdivided kinetic energy of digital economy on urban-rural integration, the GTWR model was adopted in this section for regression analysis on the four dimensions of urban-rural integration, with the basic, industrial, technical, and data kinetic energy in the index system of the digital economy as the core explanatory variables. A total of 16 (4 × 4) regression models and their results were obtained, with the adjusted R2 of all 16 models greater than 0.80. These models passed the 10% significance test, signifying a strong explanatory power of the model. The estimated coefficients are reflected in detail in Table 6.

According to Table 6, the basic kinetic energy of the digital economy had a positive influence on the four dimensions of urban-rural integration from the perspective of both the median and mean values, indicating that the key support for the digital economy to drive urban-rural integration at the current stage lies in the digital equipment basis and user basis. In rural areas, the construction of new infrastructure, including broadband, 4G/5G base stations, and intelligent agriculture, needs to be strengthened in addition to the perfect traditional hardware facilities, including transportation as well as post and telecommunications. Besides, the training of rural residents needs to be strengthened to improve the popularization rate of digital technologies. Secondly, the mean values for the influences of industrial and technical kinetic energy on the equalization of urban and rural public services were negative, mainly concentrated in central and west China. However, the proportions of positive coefficients were only 51.11% and 31.11%, respectively, the lowest among all models. Therefore, in the above-mentioned digital economy-driven urban-rural integration, the “digital divide” in the equalization of urban and rural public services mainly results from digital industrial and technical kinetic energy.

## 6. Conclusions, Implications, and Future Research

This paper elaborates on the intrinsic mechanism of the digital economy in driving the four dimensions of urban-rural integration. On this basis, the digital economy and the four dimensions of urban-rural integration in 30 provinces (municipalities and autonomous regions) in China from 2011 to 2019 were measured, and the spatial effects of the digital economy in driving urban-rural integration were empirically analyzed using the GTWR model. The main conclusions are as follows: (1) the digital economy and the four dimensions of urban-rural integration are steadily advancing with a positive trend, and the digital economy has a larger annual growth rate than urban-rural integration. The convergence of urban and rural resident consumption shows a relatively higher value among the four dimensions of urban-rural integration. (2) There is a significant spatial autocorrelation between the digital economy and the four dimensions of urban-rural integration, with the influence gradually increasing with time. (3) The digital economy exerts significantly different effects on the four dimensions of urban-rural integration. It positively impacts the equivalent allocation of urban and rural factors, integration of urban-rural three industries, and convergence of urban and rural resident consumption. At the same time, it has an inhibitory effect on the equalization of urban and rural public services in nearly half of the research areas. From the perspective of specific distribution, the equivalent allocation of urban and rural factors driven by the digital economy is characterized by the “circle-layer” declining trend of high value from inside to outside and from east to west, with Henan and Hubei as the center. The influences on the other three dimensions are characterized by the ladder characteristics of “high in the east and low in the west.” (4) After further decomposition of the digital economy, it is concluded that the digital basic kinetic energy (including digital equipment basis and user basis) has the greatest positive impact on the four dimensions of urban-rural integration, and the inhibitory effects of digital industrial kinetic energy and technological kinetic energy on the equalization of urban and rural public services are greatly affected by the “digital divide.”

The following suggestions are drawn based on the above conclusions: (1) The digital foundation should be strengthened to accelerate the extension of urban digital facilities to rural areas. The basic kinetic energy of the digital economy has the greatest impact on the four dimensions of urban-rural integration, so it is a priority to accelerate the construction of fixed broadband networks and mobile communication base stations in rural areas. Besides, practical construction funds and technical assistance should be provided to improve the quality and speed of the internet and reduce the cost of using broadband and traffic, rendering key support for the practical application of urban-rural integration, such as intelligent agricultural machinery, intelligent logistics, online education, and telemedicine. As a result, the spontaneous digital use by residents, overall user penetration rate, and the sense of gain and happiness of the people will be effectively enhanced. (2) The “digital divide” should be bridged to promote the construction of the digital ecosystem in central and western China. The problem of the “digital divide” among regions occurs in the driving of the four dimensions of urban-rural integration by the digital economy. It can be seen as the risk of the digital economy. The “access divide” can be made up by coordinating digital equipment basis among regions, while the “use divide” and “capacity divide” require long-term cross-regional talent and technology exchanges to absorb advanced digital application experience and enhance the digital use skills of people in relatively undeveloped regions. Through the continuous in-depth promotion of digital technologies and internet applications, the “digital divide” will be constantly bridged and gradually transformed into “digital dividends,” thereby achieving the coordinated development of the four dimensions of urban-rural integration among regions. (3) The weakness in integration should be mitigated, i.e., the equalization level of urban and rural public services should be improved. The weakness of the four dimensions of urban-rural integration driven by the digital economy lies in the equalization of urban-rural public services. In addition to strengthening the digital infrastructure, the development of digital industries and the research and development of digital technologies in the digital economy plays a key role in enhancing the equalization of urban-rural public services. In addition, it is necessary to continue to expand the application scenarios of digital technologies and carry out pilot projects of digital technology innovation in the fields of medical care, education, and elderly care, and continuously narrow the gap between the equalization of urban-rural public services and the other three dimensions.

In addition, the influence of the digital economy on urban-rural integration is characterized by regional heterogeneity. In the process of national economic development, it is necessary to combine the development status of various regions and adapt to local conditions. East China should continue to maintain its absolute advantage and continue to improve the level of digital economy development. At the same time, we need to provide the necessary human resources, technology, and financial support to the central and western regions to promote the coordinated development of integrated urban and rural areas among regions. For central China, it should weaken the “siphon effect” of cities on rural areas and narrow the digital divide between urban and rural areas. For west China, it is necessary to strengthen investment in digital infrastructure and the development of digital technology while avoiding the phenomenon of the urban-rural divide in developing the digital economy and promoting the coordinated development of urban-rural integration. Furthermore, for nationwide China, exchanges and cooperation between the government, market, and social entities in the regions can be strengthened, typical regional experiences can be promoted, the regional cooperation mechanism for the development of the digital economy between regions can be improved, the role of government macro-regulation can be strengthened, and the industry barriers and geographical restrictions on new models and business models in the development of the digital economy can be broken down, so as to provide certain guarantees for the synergistic development of urban-rural integration in the regions.

This research has the following implications for the whole country’s economy. First, it is conducive to promoting the economic cycle between urban and rural areas. The urban-rural economic cycle is an important aspect of the larger domestic economic cycle. The digital economy has driven high-quality factors and consumer goods to the countryside, organically connecting production and consumption, digitally upgrading the entire chain of production, distribution, distribution, and consumption of agricultural products, and making the urban-rural economic cycle smoother. Secondly, it is conducive to narrowing the gap between urban and rural areas and achieving common prosperity. Common prosperity is not only about material prosperity but also exists in many areas, such as spiritual culture, public prosperity, and social environment. The urban-rural gap also exists in these fields. On the road to common prosperity, China’s economy can further achieve high-quality development and sustainable and balanced development.

Although this study has made some progress in the development of urban-rural integration driven by the digital economy, it also has some limitations, which are as follows: First of all, there is the problem of missing statistical yearbook data in some provinces of China, such as incomplete data in Tibet and Taiwan, which cannot be counted in the empirical research; secondly, the possible obstacles in the process of digital economy-driven urban-rural integration are also worth exploring, and it can be studied in future researches.

## Figures and Tables

**Table 1:** Evaluation index system of urban-rural integration.

First-Grade Index	Second-Grade Index	Third-Grade Index
Equivalent allocation of urban and rural factors	Capitalal location	Average fixed asset investment ratio of the urban and rural labor force
		Agricultural, forestry, and water fiscal expenditure/total fiscal expenditure
	Labor force allocation	Ratio of urban and rural human capital
		Ratio of people leaving the registered residence for half a year
	Technology allocation	Science and technology expenditure/total fiscal expenditure
		Number of agricultural technicians/total rural population
	Land allocation	Cultivated land area/built-up area
		Built-up area/land area
Integration of three industries in urban and rural areas	Industry chain integration	Total output value of agricultural and sideline products processing industry/ the total output value of agricultural industries
		Agricultural mechanization degree/cultivated land area
		Number of employees in rural secondary and tertiary industries/ number of rural employees
	Multi- functional integration	Per capita cultural tourism expenses
		Number of employees in the cultural tourism industry/total number of employees
Equalization of urban and rural public services	Medical equalization	Per capita fiscal expenditure on medicine
		Ratio of beds in medical and health institutions per 1000 people in urban and rural areas
		Ratio of health technicians per 1000 population in urban and rural areas
	Educational equalization	Per capita fiscal expenditure on education
		Ratio of urban and rural secondary school entrance rate
		Ratio of urban and rural high school entrance rate
	Social security equalization	Per capita fiscal expenditure on social security
		Coverage rate of urban and rural endowment insurance
		Coverage rate of urban and rural medical insurance
		Per capita fiscal expenditure on general public services
Convergence of urban and rural resident consumption	Resident income	Per capita disposable income
		Ratio of urban and rural disposable income
	Resident consumption	Per capita consumption expenditure
		Ratio of per capita urban and rural consumption expenditure
		Ratio of urban and rural Engel coefficients
		Ratio of per capita urban and rural education expenditure
		Ratio of per capita urban and rural medical expenditure

**Table 2:** Evaluation index system of the digital economy.

First-Grade Index	Second-Grade Index	Third-Grade Index
Basic kinetic energy	Equipment basis	Length of fiber cable line of land units
		Internet broadband access port
	User basis	Mobile phone popularization rate
		Number of mobile internet users
Industrial kinetic energy	Digital industrialization	Total number of telecom services
		Total output value of the digital industry (manufacturing industry)
		Total output value of the digital industry (service industry)
	Industrial digitalization	E-commerce sales
		Digital inclusive finance
Technical kinetic energy	Technical input	Full-time equivalent of high-tech R&D personnel
		Number of high-tech R&D projects
	Technical output	Technical market turnover
		Number of effective patents in high-tech industries
Data kinetic energy	Data readiness	Number of listed big data enterprises
		Total market value of listed big data enterprises
	Data openness	Number of data exchange centers

**Table 3:** Bivariate Global Moran’s I statistics of the digital economy and urban-rural integration.

	Digital Economy- Equivalent Allocation of Urban and Rural Factors	Digital Economy-Integration of Three Industries in Urban and Rural Areas	Digital Economy-Equalization of Urban and Rural Public Services	Digital Economy-Convergence of Urban and Rural Resident Consumption
2011	0.072 (1.5576) *	0.118 (2.3221) **	0.063 (1.1295)	0.142 (2.9027) ***
2015	0.080 (1.6734) *	0.127 (2.4816) ***	0.106 (1.5919) *	0.196 (3.8723) ***
2019	0.152 (2.2904) **	0.126 (2.4662) ***	0.134 (2.3121) **	0.206 (4.0451) ***

* Notes: Figures inside the brackets are Z test values, * *p* < 0.1, ** *p* < 0.05, and *** *p* < 0.01.

**Table 4:** Description of variables.

Variable Type	Index	Explanatory Variable	Description
Explained variable	Equivalent allocation of urban and rural factors	fac_alloca	The urban-rural integration level of each dimension calculated according to the above-mentioned analysis
	Integration of three industries in urban and rural areas	three_indus	
	Equalization of urban and rural public services	pub_serve	
	Convergence of urban and rural resident consumption	resid_consum	
Core explanatory variable	Digital economy	digital	Score of the development level of the digital economy obtained
Control variable	Economic development	gdp	GDP
	Opening up	open	Value of import and export
	Government supplies	gov	Government expenditure
	Natural conditions	topogra	Relief amplitude

**Table 5:** Results of all kinds of weighted regression analysis.

Explanatory Variable		GTWR			GWR			TWR		
		RSS	Sigma	Adj_R2	RSS	Sigma	Adj_R2	RSS	Sigma	Adj_R2
(1)	fac_alloca	0.340	0.035	0.872	0.558	0.045	0.788	1.104	0.064	0.581
(2)	three_indus	0.672	0.049	0.901	0.722	0.051	0.892	3.040	0.106	0.546
(3)	pub_serve	0.206	0.027	0.961	0.263	0.031	0.950	0.556	0.045	0.897
(4)	resid_consm	0.934	0.058	0.854	1.291	0.069	0.795	3.592	0.115	0.430

**Table 6:** Spatial effect decomposition of the digital economy.

Explanatory Variable			Explained Variable			
Digital Economy Decomposition		GTWR Estimated Coefficient	Equivalent Allocation of Urban and RuralFactors	Integration of Three Industries in Urban and Rural Areas	Equalization of Urban and Rural Public Services	Convergence of Urban and Rural Resident Consumption
Digital economy	Basic Kinetic energy	Coefficient range	{−0.985, 2.397}	{−1.465, 2.186}	{−1.078, 0.787}	{−1.880, 1.913}
		Median	0.6635	0.9335	0.0506	0.3362
		Mean	0.6887	0.6413	0.0221	0.1755
		Proportion of positive values	97.41%	73.71%	75.56%	72.96%
	Industrial kinetic energy	Coefficient range	{−2.716, 1.898}	{−1.317, 1.768}	{−6.088, 1.584}	{−1.834, 0.853}
		Median	0.5955	0.8237	0.0014	0.2623
		Mean	0.4102	0.6020	−0.2593	0.2467
		Proportion of positive values	85.19%	84.81%	51.11%	69.25%
	Technical kinetic energy	Coefficient range	{−0.825, 1.470}	{−1.985, 1.886}	{−2.781, 0.567}	{−3.574, 1.878}
		Median	0.5227	0.6521	−0.0495	0.1192
		Mean	0.4633	0.5287	−0.1245	0.0641
		Proportion of positive values	72.22%	67.03%	31.11%	54.44%
	Data kinetic energy	Coefficient range	{−1.928, 0.840}	{−0.511, 0.782}	{−0.699, 2.391}	{−1.071, 0.617}
		Median	0.3553	0.5012	0.0187	0.1507
		Mean	0.2783	0.3349	0.0124	0.0728
		Proportion of positive values	92.97%	77.78%	63.70%	70.00%

## Data Availability

Available upon request by contacting the author.
